# CCHCR1 Is Up-Regulated in Skin Cancer and Associated with EGFR Expression

**DOI:** 10.1371/journal.pone.0006030

**Published:** 2009-06-24

**Authors:** Sari Suomela, Outi Elomaa, Tiina Skoog, Risto Ala-aho, Leila Jeskanen, Jenita Pärssinen, Leena Latonen, Reidar Grénman, Juha Kere, Veli-Matti Kähäri, Ulpu Saarialho-Kere

**Affiliations:** 1 Department of Dermatology, Helsinki University Central Hospital and Biomedicum Helsinki, University of Helsinki, Helsinki, Finland; 2 Department of Pathology, Helsinki University Central Hospital and Biomedicum Helsinki, University of Helsinki, Helsinki, Finland; 3 Department of Medical Genetics, University of Helsinki, and Folkhälsan Institute of Genetics, Helsinki, Finland; 4 Molecular Cancer Biology Program and Haartman Institute, University of Helsinki, Helsinki, Finland; 5 Department of Dermatology, University of Turku, Turku University Central Hospital, Turku, Finland; 6 Medicity Research Laboratory, University of Turku, Turku, Finland; 7 Department of Otorhinolaryngology-Head and Neck Surgery, Turku University Central Hospital, Turku, Finland; 8 Department of Biosciences and Nutrition at Novum, Karolinska Institutet, Huddinge, Sweden; 9 Departments of Clinical Science and Education and Section of Dermatology, Karolinska Institutet at Stockholm Söder Hospital, Stockholm, Sweden; Harvard Institute of Medicine, United States of America

## Abstract

Despite chronic inflammation, psoriatic lesions hardly ever progress to skin cancer. Aberrant function of the CCHCR1 gene (Coiled-Coil α-Helical Rod protein 1, HCR) within the PSORS1 locus may contribute to the onset of psoriasis. As CCHCR1 is expressed in certain cancers and regulates keratinocyte (KC) proliferation in a transgenic mouse model, we studied its relation to proliferation in cutaneous squamous cell cancer (SCC) cell lines by expression arrays and quantitative RT-PCR and in skin tumors by immunohistochemistry. CCHCR1 protein was detected in the pushing border of SCC and lining basal cell carcinoma islands. Different from psoriasis, Ki67 had a similar expression pattern as CCHCR1. The most intense CCHCR1 staining occurred in areas positive for epidermal growth factor receptor (EGFR). Expression of CCHCR1 mRNA was upregulated 30–80% in SCC lines when compared to normal KCs and correlated positively with Ki67 expression. The most aggressive and invasive tumor cell lines (RT3, FaDu) expressed CCHCR1 mRNA less than non-tumorigenic HaCaT cells. Moreover, the tumor promoters okadaic acid and menadione downregulated CCHCR1 mRNA. We conclude that both in psoriasis and the early stages of KC transformation, CCHCR1 may function as a negative regulator of proliferation, but beyond a certain point in oncogenesis cannot control this phenomenon any longer.

## Introduction

We have previously shown that the CCHCR1 gene within the major psoriasis susceptibility locus PSORS1 may function as a negative regulator of KC proliferation [1]–[3]. The CCHCR1 gene encodes a 782 amino acid protein (GenBank AY029160) [4] that is differently expressed in lesional psoriatic skin compared to normal skin: lesionally CCHCR1 locates in basal and suprabasal KCs where the epidermis is at its thinnest. Staining for the cell proliferation marker Ki67 shows inverse correlation to CCHCR1 in psoriasis lesions, consistent with a role in KC proliferation [1], [4]. Furthermore, our mouse model overexpressing the CCHCR1*WWCC psoriasis risk allele under keratin 14 promoter shows impaired proliferation capability of KCs [3], suggesting less active activator protein 1 (AP-1) mediated signaling in the risk mice. AP-1 mediated signaling regulates genes associated with cell proliferation, including EGFR also known as ErbB1 [5].

Psoriasis lesions hardly ever progress to skin cancer despite chronic inflammation that often is a risk factor for cancer [6], [7]. Both psoriasis and skin cancer are characterized by uncontrolled KC proliferation in addition to angiogenesis and inflammation. Cellular proliferation and differentiation can be mediated via EGFR signaling [8]. Multiple EGFR ligands (TGF-α, amphiregulin, heparin-binding EGF-like growth factor) are overexpressed in psoriasis lesions, and transgenic expression of the human amphiregulin gene induces a psoriasis-like phenotype [8]. EGFR signaling has also been implicated in the pathogenesis of non-melanoma skin cancer [8]–[10]. Interestingly, both improvement and exacerbation of psoriasis have been reported in association with EGFR tyrosine kinase inhibitor cancer treatment [11], [12].

In our previous studies, CCHCR1 expression was detected in nonproliferating cells in breast and lung adenocarcinomas: the hyperproliferation marker Ki67 was detected in adjacent, but not in the same cells as CCHCR1 [1]. Furthermore, EGF up-regulated CCHCR1 protein expression in HaCaT cells [2]. To further understand the function of CCHCR1 in KC transformation and skin cancer, we studied its expression in premalignant and malignant squamous lesions and basal cell carcinoma (BCC). As EGFR and its downstream target cyclin-D1 are involved in cancer development and possibly affected by the putative CCHCR1 pathways, we examined their expression in comparison to CCHCR1. The relation of CCHCR1 to KC hyperproliferation was highlighted by staining adjacent samples with the Ki67 antibody. We also studied the correlation of CCHCR1 expression level to those of Ki67 and EGFR in cultures of cells with different proliferative and invasive characteristics. CCHCR1 mRNA levels were determined in HaCaT cultures incubated with diverse bioactive agents with importance in tumor promotion, oxidative stress or psoriatic inflammation. Finally, the expression levels of CCHCR1, Ki67, and EGFR mRNAs were determined in an oligonucleotide microarray of cutaneous SCC cell lines and normal human epidermal KC cell lines. Our results suggest that CCHCR1 is expressed in non-melanoma skin cancers in association with EGFR and Ki67 in vivo. However, in cell culture the most atypic cells express CCHCR1 less than immortalized HaCaT cells, and tumor promotion and induced proliferation of HaCaT cells correlates with reduced CCHCR1 expression.

## Results

### CCHCR1 is expressed in SCC in association with EGFR, cyclin-D1, and Ki67

**Figure 1:**
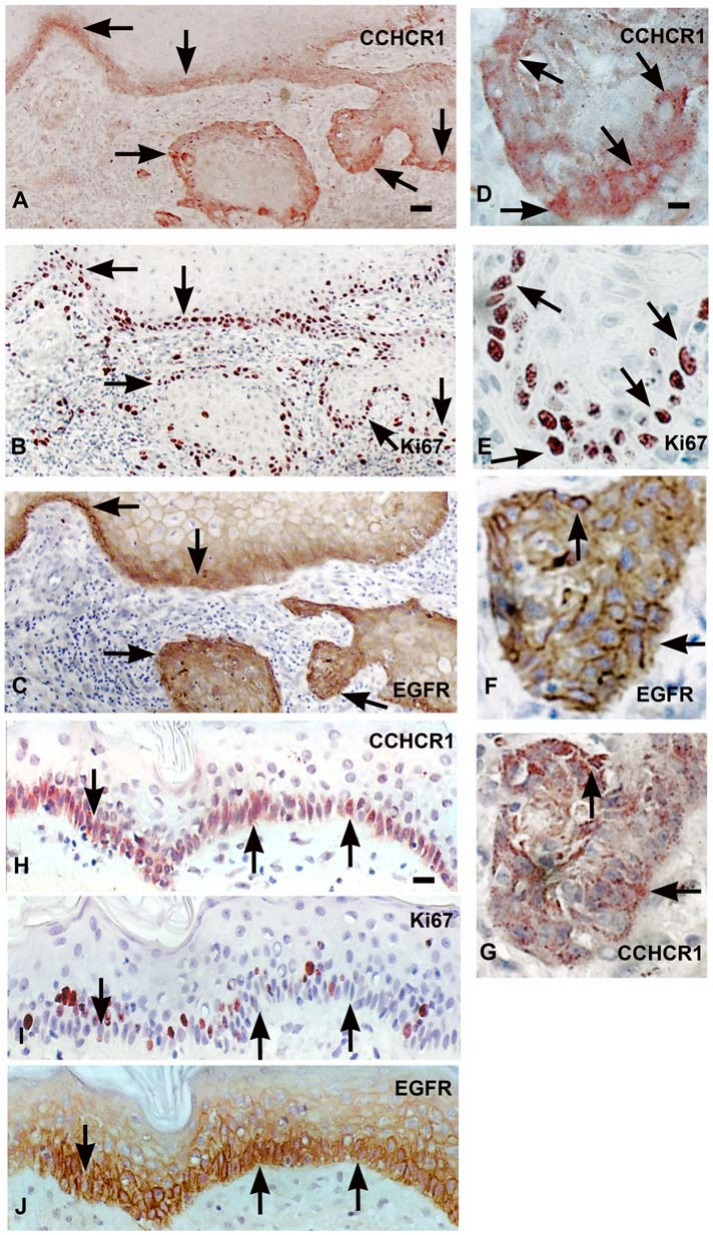
CCHCR1, Ki67, and EGFR are coexpressed in grade I SCC and in normal skin. Serial sections of grade I SCC (A–C) and normal skin (H–J) were immunostained with antibodies against CCHCR1, Ki67, and EGFR. Higher magnifications of A–C are also shown (D–F, respectively). CCHCR1 staining (G) in an adjacent section to EGFR staining (F). CCHCR1 protein (A) is expressed in proliferative cancer cells at the invasive front of dermal cancer cell islands of an SCC in association with the hyperproliferation marker Ki67 (B) and EGFR (C). The Ki67 positive cells (E) express CCHCR1 (D). EGFR staining (F) associates with CCHCR1 staining (G) in adjacent sections. Normal skin samples express CCHCR1 (H) and EGFR (J) in basal KCs, while Ki67 expression (I) is more sparse. Arrows point at illustrative positions. *Scale bars:* (A–C) 50 µm; (D–G) 12.5 µm; (H–J) 25 µm.

**Figure 2:**
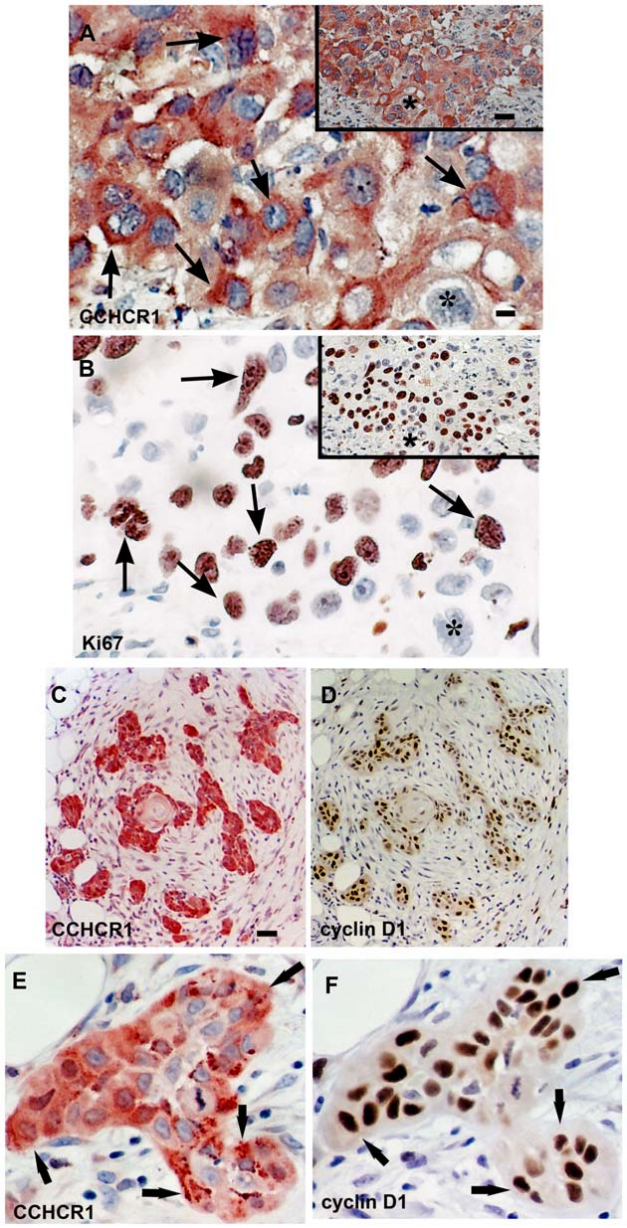
Heterogeneous expression of CCHCR1, Ki67, and cyclin D1 in grade III and II SCCs. Serial sections of grade III (A, B) and grade II (C, D) SCC were stained with the indicated antibodies. Higher magnification of another grade II SCC (E, F). In grade III SCC (A, B), expression of CCHCR1 is heterogeneous: many, but not all, cancer cells express both CCHCR1 (A) and Ki67 (B). The insets show lower magnification of the same region, asterisk points out a cancer cell with negative CCHCR1 and Ki67 expression. In grade II SCC (C, D), infiltrative outgrowth is CCHCR1 positive (C), and the CCHCR1 positive cancer nests are also cyclin-D1 positive (D). Adjacent sections of grade II SCC (E, F) show association of CCHCR1 and cyclin-D1 positive cells. Arrows indicate corresponding areas. *Scale bars:* (A,B,E,F) 12.5 µm; (C,D, insets) 50 µm.

To study the role of CCHCR1 in KC proliferation and transformation, we investigated CCHCR1 protein expression by immunohistochemistry in different skin tumors in relation to EGFR, its downstream target cyclin-D1, and the hyperproliferation marker Ki67. CCHCR1 positive cells were present in 20/22 SCCs studied. Proliferative cancer cells at the invasive front expressed CCHCR1 (Figure 1A,D,G, S1; Figure 2A,C,E), whereas the cohesive cancer cells in the middle were CCHCR1 negative. Infiltrative outgrowth was typical of CCHCR1 positive areas, however, the atypic cells were heterogenous for CCHCR1 expression (Figure 2A). CCHCR1 expression in SCC was associated with positive EGFR staining in all specimens (Figure 1A,C,D,F). The CCHCR1 positive cancer nests in the lower dermis were cyclin-D1 positive as well (Figure 2C–F). Cells positive for the hyperproliferation marker Ki67 were located mostly in the same areas as CCHCR1 positive cells (Figure 1B,E; 2B). However, in grade III SCCs Ki67 was more abundantly present also in cohesive tumor areas that were devoid of CCHCR1 expression (data not shown). For comparison, a normal skin sample is shown with basal KCs positive for CCHCR1 and EGFR, and scattered positivity for Ki67 (Figure 1H–J).

### CCHCR1 is expressed by the palisading cancer cells in BCC

**Figure 3:**
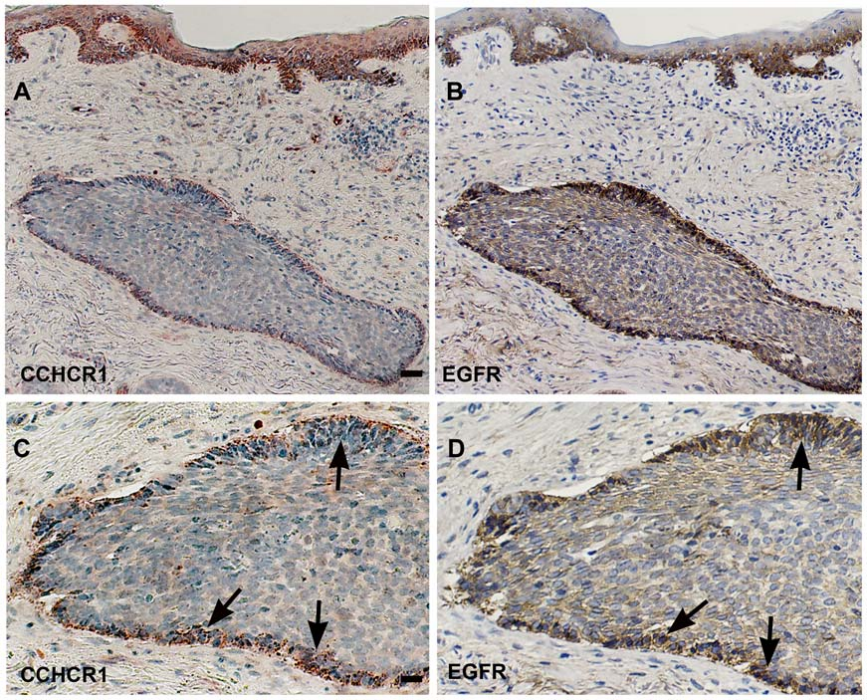
CCHCR1 and EGFR colocalize in BCC. Serial sections of BCC (A, B) were stained with antibodies against CCHCR1 and EGFR. Higher magnifications of A and B are also shown (C, D, respectively). CCHCR1 protein is expressed in a granular pattern in the cytoplasm of the palisading cancer cells of a nodular BCC (A, C). CCHCR1 expression (C) co-localizes with EGFR staining (D). CCHCR1 and EGFR are also expressed in basal KCs (A, B). Arrows show corresponding positions. *Scale bars:* (A,B) 50 µm; (C,D) 25 µm.

In BCC, CCHCR1 was expressed especially in the cytoplasm of the palisading cancer cells of the well-defined carcinoma islands (Figure 3A,C). Three out of 15 BCC specimens were sclerosing, but CCHCR1 was not more abundant in them (data not shown). CCHCR1 was expressed in a granular pattern in seven samples (Figure 3A,C) co-localizing with EGFR (Figure 3B,D). All five samples studied were cyclin-D1 positive: however, its expression was not as strong as that of CCHCR1 and more concentrated to the centre of the tumor nests (data not shown). Cytoplasmic expression of CCHCR1 in basal KCs in normal looking skin adjacent to the BCC area varied between samples. EGFR expression was higher in basal KCs of normal skin than in palisading cells in most of the samples.

**Figure 4:**
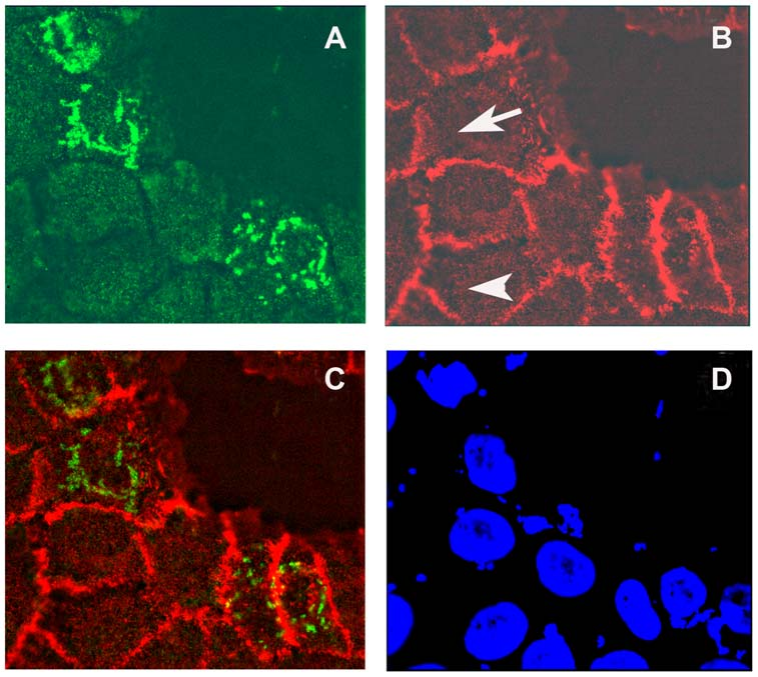
Immunofluorescence staining of HaCaT cells for CCHCR1 and EGFR. CCHCR1 transfected HaCaT cells (A–D) stained with antibodies against CCHCR1 (A) and EGFR (B). Overlay of A and B is shown in (C), and DAPI staining showing nuclei in (D). Cells expressing CCHCR1 (green) were also positive for EGFR (red) but the two proteins did not co-localize (C). CCHCR1 positive (arrow) and negative (arrow head) cells exhibit similar EGFR expression suggesting that CCHCR1 overexpression does not affect EGFR expression (B).

To study whether CCHCR1 and EGFR co-localize also at the cellular level, we transiently transfected HaCaT cells with a CCHCR1 construct and visualized CCHCR1 and EGFR expression with immunofluorescence. CCHCR1 expression was detected as a ring-like, cytoplasmic pattern, while EGFR expression was more or less membrane bound and did not co-localize with CCHCR1 staining (Figure 4A–D). Transfected and non-transfected cells showed similar EGFR expression, suggesting that CCHCR1 does not influence EGFR expression or localization (Figure 4B).

### CCHCR1 expression in keratoacanthomas is associated with lymphocyte infiltration

**Figure 5:**
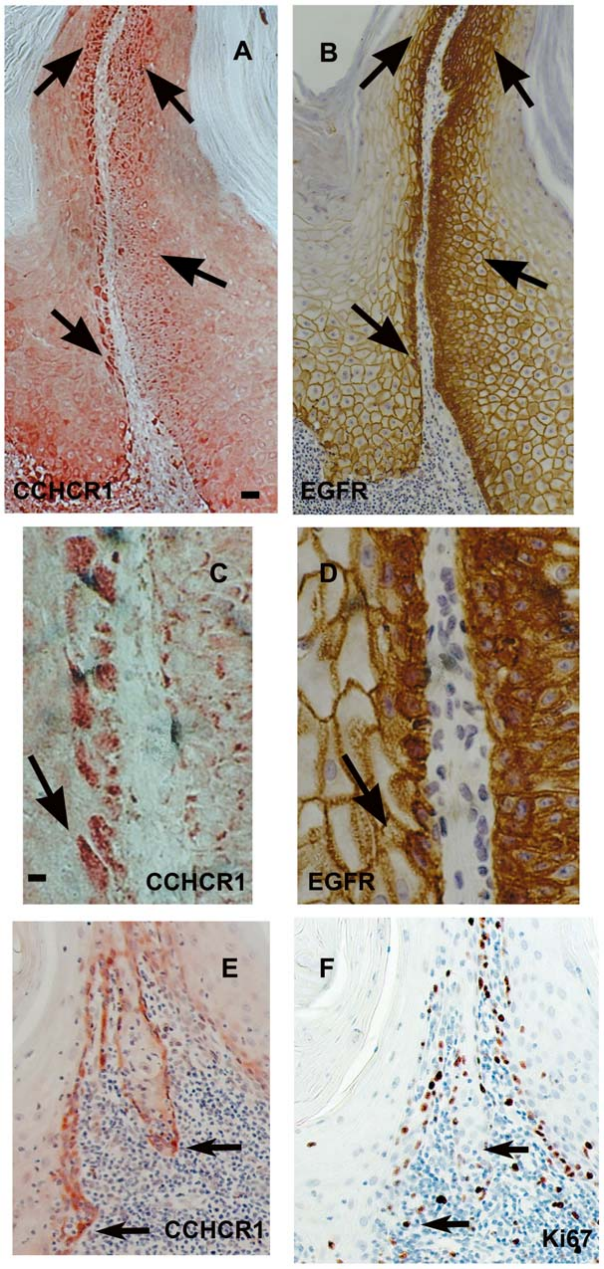
Expression of CCHCR1, EGFR, and Ki67 in keratoacanthoma. Serial sections of KA (A, B, E, F) were stained with the antibodies against CCHCR1 and EGFR (A, B) or against CCHCR1 and Ki67 (E, F). Higher magnifications of A and B are shown (C, D, respectively). The pushing border of a KA is CCHCR1 positive (A) co-localizing with EGFR (B). Another sample is shown (E) with a prominent lymphocyte infiltrate and cytoplasmic CCHCR1 expression of the pushing border cells. More scarce Ki67 expression (F) is defined to the same areas but not necessarily to the same cells. Arrows indicate corresponding positions. *Scale bars:* (A, B, E, F) 50 µm; (C, D) 12.5 µm.

Keratoacanthoma (KA), histologically a malignant neoplasm but behaving in a benign manner, is often considered a “precursor” lesion to SCC. KAs generally showed positive CCHCR1 staining especially in KCs of the pushing border in areas with a prominent lymphocyte infiltrate surrounding the tumor (Figure 5A,C). EGFR-positive cells were present in the same areas in the studied samples (Figure 5B,D). In the CCHCR1 positive area, Ki67 expression was also encountered in all three samples, but with less intensity than in SCCs (Figure 5E,F).

### Bowen's disease and actinic keratoses express CCHCR1 in spongiotic and inflammatory areas

**Figure 6:**
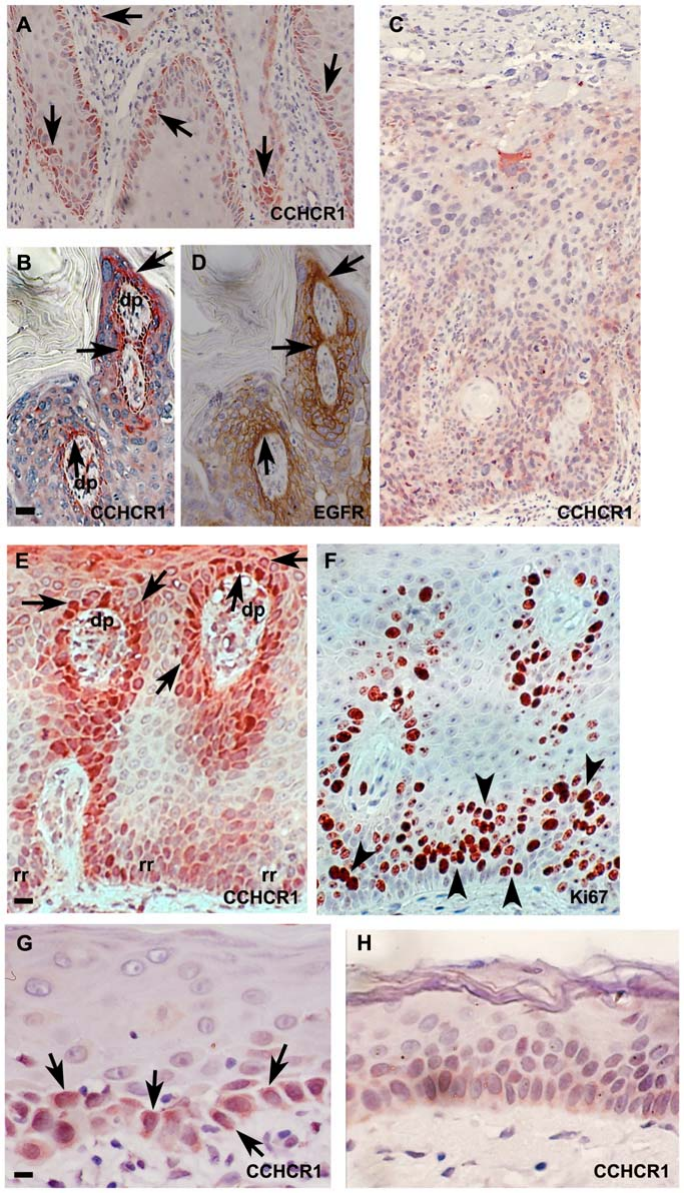
Expression of CCHCR1 in Bowen's disease, psoriasis, and actinic keratosis. Sections of Bowen's disease (A–D) immunostained with antibodies against CCHCR1 and EGFR as indicated. Serial sections of psoriasis (E, F) were stained with antibodies for CCHCR1 and Ki67. AK samples (G, H) were stained with CCHCR1 antibodies. In Bowen's disease (A, B), spongiosis, inflammatory infiltrate, and capillary proliferation are associated with CCHCR1 expression while areas lacking inflammation and spongiosis express less CCHCR1 (C). EGFR expression (D) in basal and suprabasal KCs of dermal papillae associates with CCHCR1 expression (B). Also in psoriasis (E), CCHCR1 is expressed basally or suprabasally (arrows) overlying the dermal papillae (dp) while rete ridges (rr) projecting into the dermis are almost negative for CCHCR1. In contrast, Ki67 expression (F) localizes to regions that are almost negative for CCHCR1 cells (arrowheads). AK samples (G) express CCHCR1 basally and suprabasally in association with KC heterogeneity, spongiosis or inflammation but adjacent areas with less inflammation or spongiosis (H) stain only faintly. *Scale bars:* (A–D) 50 µm; (E, F) 25 µm; (G, H) 12.5 µm.

Spongiosis, inflammatory infiltrate, and capillary proliferation were associated with CCHCR1 expression in 11/21 Bowen's disease (SCC in situ) samples (Figure 6A,B). Areas lacking inflammation and spongiosis expressed less CCHCR1 (Figure 6C), neither was CCHCR1 expression associated with KC atypia. In Bowen's disease, cytoplasmic CCHCR1 staining was encountered basally and suprabasally in dermal papillae (Figure 6B) resembling CCHCR1 staining of psoriatic lesional skin (Figure 6E), especially in hypertrophic samples. Also EGFR expression in Bowen's disease resembled the expression pattern of CCHCR1 (Figure 6D). In psoriasis, CCHCR1 expression was most intense in areas with less Ki67 positive KCs (Figure 6F). Eight of 11 studied actinic keratosis (AK) specimens had CCHCR1 positive basal or suprabasal KCs (Figure 6G). CCHCR1 positive cells were often encountered in regions with spongiosis or inflammation.

### Expression of CCHCR1 is up-regulated and correlates with Ki67 expression in cutaneous SCC cell lines

**Figure 7:**
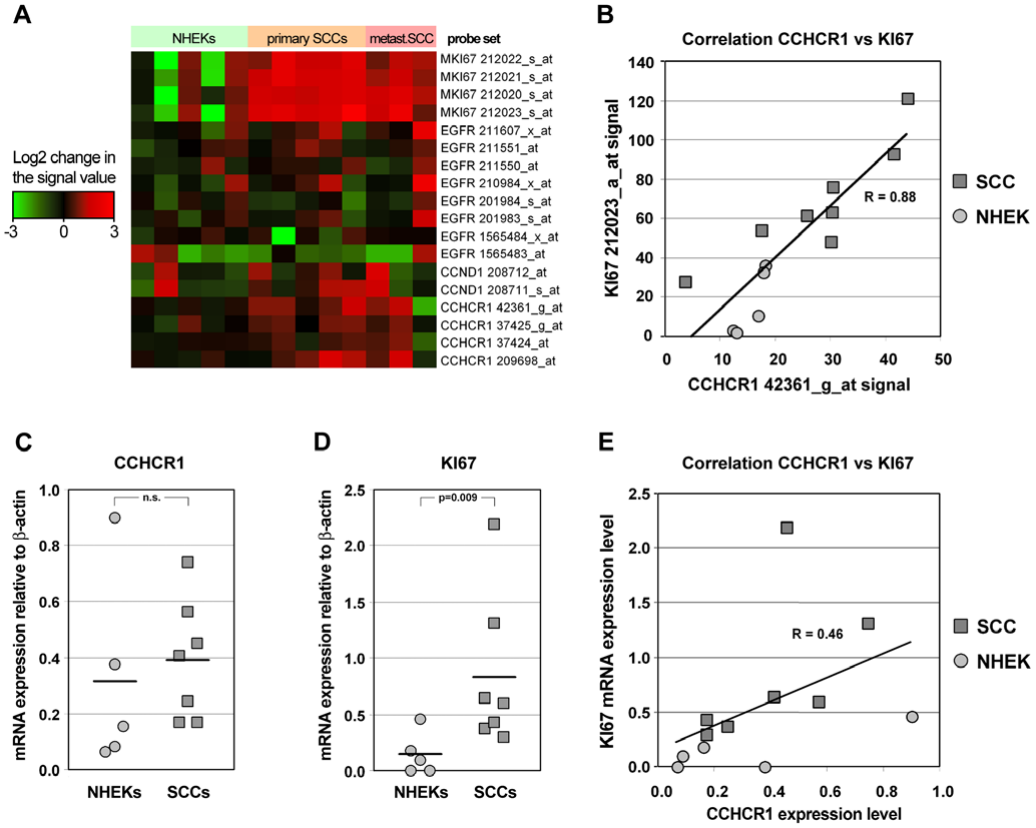
CCHCR1 mRNA expression in normal keratinocytes and SCC cell lines correlates with Ki67 expression. A) Ki67, EGFR, cyclin-D1 and CCHCR1 gene expression profile of five normal epidermal KC and eight cutaneous SCC cell lines (heatmap). Signal values of the probe sets were compared to the mean signal values of each probe set in KCs. The colouring is based on the log2 values of the change in the signal values. The up-regulated genes are shown in red and down-regulated genes are shown in green. B) Correlation between CCHCR1 and Ki67 probe sets was calculated between the signal values of one CCHCR1 and one Ki67 probe set in the HG-U133 Plus 3.0 array. Pearson's correlation coefficient R = 0.88. C) CCHCR1 and D) Ki67 mRNA expression levels in the normal KCs and cutaneous SCC cell lines as measured by qRT-PCR (TaqMan). Expression levels of CCHCR1 and Ki67 in the normal KCs and SCC cell lines were analyzed by qRT-PCR and corrected for the β-actin mRNA levels in the same samples. E) Correlation between CCHCR1 and Ki67 probe sets was calculated between the signal values of CCHCR1 and Ki67 mRNA levels. Pearson's correlation coefficient R = 0.46.

Next, we compared the mRNA expression profiles of CCHCR1, EGFR, Ki67, and cyclin-D1 in different cutaneous SCC cell lines (five primary and three metastatic), by performing Affymetrix experiments. The oligonucleotide microarray based expression profile showed that CCHCR1 gene is expressed in SCC cells (Figure 7A). Signal levels of the CCHCR1 probe sets were up to 80% higher in the cutaneous SCC cell lines (n = 8) than in the normal human epidermal KCs (n = 5), but there were no marked differences in CCHCR1 expression levels between primary and metastatic SCC lines. Expression levels in the SCC cell lines were variable, but the average levels showed a slight increase (of 30%; p>0.05) when compared to normal KCs by quantitative real-time RT-PCR (TaqMan) (Figure 7C). Signal levels of the Ki67 probe sets were 3–4 fold higher in SCC cell lines than in normal epidermal KCs (Figure 7A). This observation was also confirmed by TaqMan PCR (Figure 7D). Importantly, expression levels of CCHCR1 correlated with the levels of Ki67 in the microarray data (Figure 7B). A slight correlation (R = 0.46) between the CCHCR1 and Ki67 mRNA levels was observed in the TaqMan data as well (Figure 7E). The expression levels of EGFR mRNA in the SCC cell lines were between 80% and 180% of the normal KC expression levels in 4 of 8 probe sets (Figure 7A). Both cyclin-D1 probe sets were present in all cell lines and the mean signal levels were up to 60% higher in the SCC cell lines as compared to normal KCs. CCHCR1 had a slight negative correlation with the EGFR expression level, but no correlation between CCHCR1 and cyclin-D1 was detected (data not shown).

### Okadaic acid and menadione downregulate CCHCR1 mRNA expression in HaCaT cells

**Figure 8:**
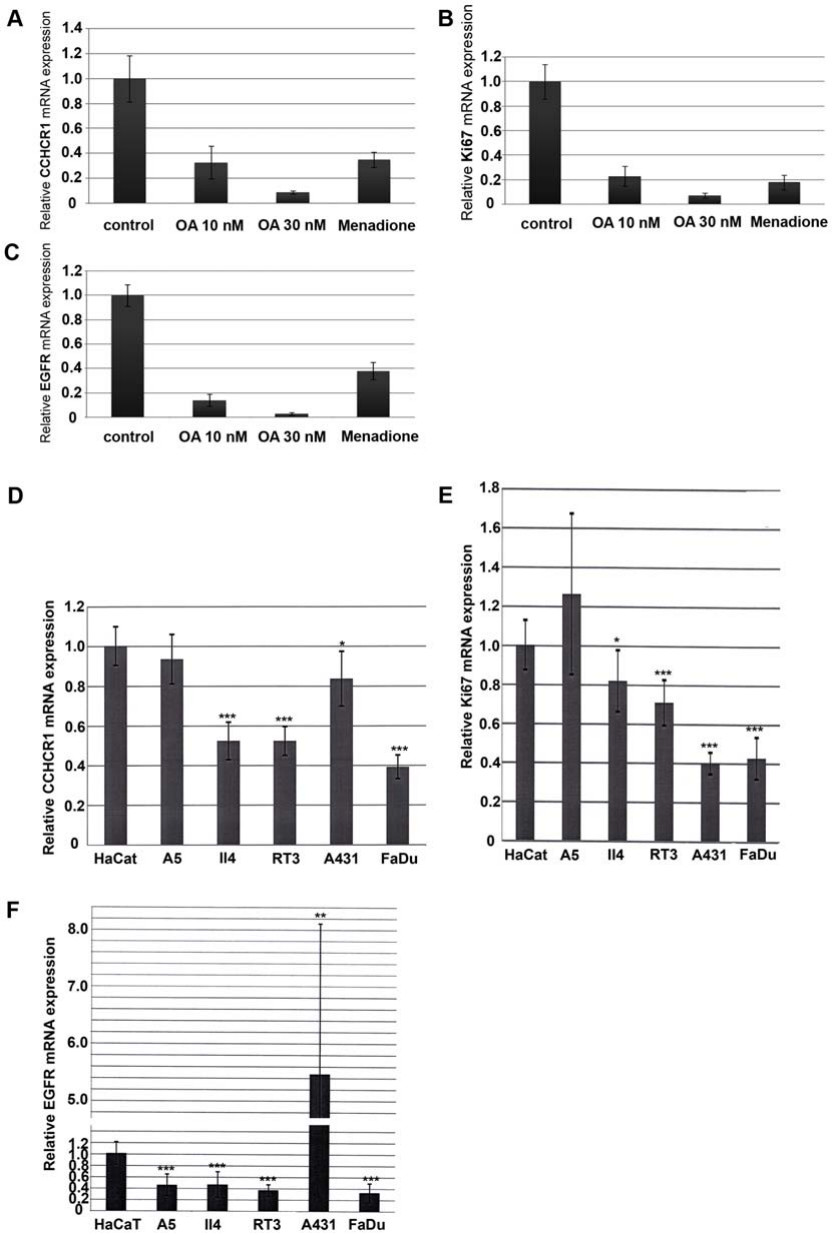
Expression and regulation of CCHCR1, Ki67, and EGFR mRNA in HaCaT cells, its ras-transformed clones and SCC cell lines. CCHCR1 (A), Ki67 (B), and EGFR (C) mRNA expression levels (TaqMan) in HaCaT cells after treatment with the tumor promoters OA and menadione. CCHCR1 (D), Ki67 (E) and EGFR (F) mRNA expression in HaCaT, A5, II4, RT3, A431, and FaDu cells. The invasive cells II4 and FaDu and metastatic RT3 cells expressed less CCHCR1 (D) and Ki67 (E) than HaCaT and A5 cells. A431 cells (F) express EGFR mRNA clearly more than other cell lines. The ras-transformed clones A5, II4, and RT3 and FaDu cells express EGFR less than HaCaT cells. Quantitative RT-PCR results are shown relative to mRNA levels from corresponding control cells (assigned the value 1). Expression levels of CCHCR1, Ki67, and EGFR in HaCaT cells were normalized to the GAPDH mRNA levels in the same samples. * p<0.05, ** p<0.01, *** p<0.001.

In order to determine whether different bioactive agents alter CCHCR1 mRNA expression, we treated HaCaT cell cultures with tumor promoters, oxidative stress inducers, and agents involved in psoriatic inflammation. Okadaic acid (OA) and menadione, both compounds shown to promote tumors in vivo and induce oxidative stress, downregulated CCHCR1 mRNA levels in HaCaT cells up to 10-fold with increasing dose (OA) and 3-fold (menadione) (Figure 8A). EGFR and Ki67 mRNA levels of OA and menadione treated HaCaT cells were downregulated as well (Figure 8B,C). Menadione or OA treatments did not essentially alter expression of the house-keeping gene GAPDH in HaCaT cells, suggesting that these agents did not affect viability of the cells. The tumor promoters 12-phorbol-13-myristate-acetate (PMA) and staurosporine or the anti-estrogen tamoxifen, H_2_O_2_ producing oxidative stress, leptin, IL-6 or staphylococcal endotoxin B, activin or anisomycin, did not significantly influence CCHCR1 mRNA levels (data not shown).

### CCHCR1 is downregulated in the most aggressive tumor cell lines

To further study the role of CCHCR1 in KC transformation, we compared CCHCR1 mRNA expression in cell lines with different metastatic and invasive properties. Based on quantitative TaqMan RT-PCR, CCHCR1 and Ki67 expression levels were similar in immortalized HaCaT and tumorigenic A5 ras-transformed cell lines (Figure 8D,E). However, with ascending tumorigenicity in ras-transformation, invasive II4 and metastatic RT3 cells expressed less CCHCR1 mRNA, and this trend was also seen with malignant A431 cells (Figure 8D). Similarly, FaDu cells that are more invasive than HaCaT and A431 cells [13] expressed less CCHCR1, Ki67, and EGFR (Figure 8D–F). Also Ki67 and EGFR expression diminished with ras-transformation (Figure 8E,F). Interestingly, A431 cells were the only cells to express EGFR significantly more than HaCaT cells (Figure 8F), Ki67 and CCHCR1 expression remaining at a lower level (Figure 8D,E).

### CCHCR1 mRNA expression is downregulated when keratinocytes are in a rapidly proliferating state

**Figure 9:**
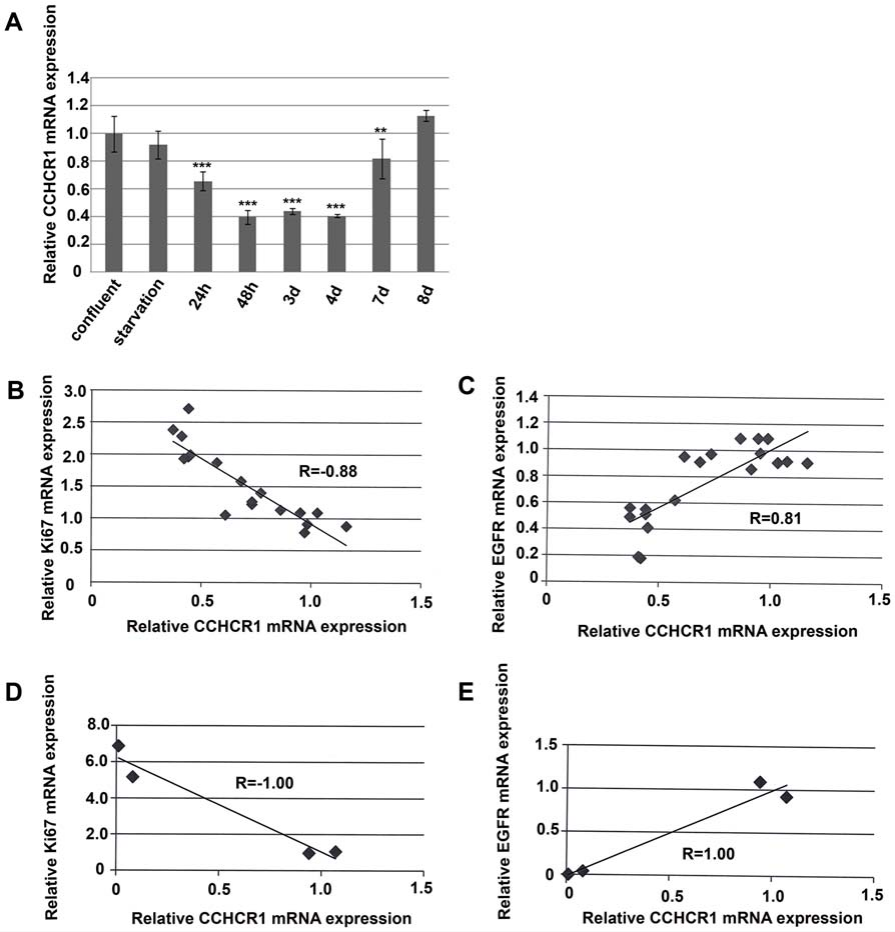
Expression of CCHCR1, Ki67, and EGFR in HaCaT cell proliferation assay. A) Relative expression of CCHCR1 mRNA (as measured by TaqMan) at different time points (from 24 h to 8 d) after releasing the cells from serum starvation and high density culturing. Expression of CCHCR1 did not differ between confluent (control) cells and quiescent (starved) cells. Control cells expressed CCHCR1 2.5-fold more than proliferative HaCaT cells (24–48 h, 3–4 d). As confluency was reattained (8 d), the expression of CCHCR1 increased to the level of the control cells. B) Correlation between relative CCHCR1 and Ki67 expression levels. When CCHCR1 mRNA expression levels were compared to those of Ki67, a negative correlation was seen, confirming the proliferative status of the HaCaT cells. C) EGFR mRNA expression correlated with CCHCR1 mRNA expression. D) Correlation between CCHCR1 and Ki67 expression in the experiment with lower cell density. The negative correlation of CCHCR1 expression with Ki67 expression was even more profound as relative CCHCR1 mRNA decreased near to zero in the two control cells. E) Correlation between CCHCR1 and EGFR expression in the experiment with lower cell density. Here again, CCHCR1 expression correlated with EGFR expression. TaqMan PCR results are shown relative to mRNA levels from corresponding control cells assigned the value 1. Expression levels of CCHCR1, Ki67, and EGFR in HaCaT cells were normalized to the GAPDH mRNA levels in the same samples. * p<0.05, ** p<0.01, *** p<0.001.

To study CCHCR1 mRNA expression in immortal but non-tumorigenic HaCaT cells of different proliferative stages, we performed cell culture experiments according to the strategy of Pivarcsi et al. [14]. In our experiments, confluent HaCaT cells (controls) and confluent cells after a 1-wk starving period (cells forced to quiescence) expressed CCHCR1 more than HaCaT cells stimulated to proliferate by addition of serum to a non-confluent subculture (Figure 9A). CCHCR1 expression decreased especially during the first four days. The proliferation status of the cells (as confirmed by Ki67 mRNA expression) correlated negatively with CCHCR1 expression (Figure 9B): decrease in CCHCR1 expression was associated with increase in Ki67 expression. As the cells reattained confluency, the expression of CCHCR1 increased again to the original level (Figure 9A). One of the four experiments was done using even lower numbers of cells. Here we demonstrated an intense proliferation during the first time points (24 h and 48 h) as relative Ki67 mRNA expression increased 6-fold compared to control cells (two sets of control cells giving the two values around value 1). The negative correlation of CCHCR1 expression with Ki67 expression was even more profound as relative CCHCR1 mRNA at the same time decreased near to zero from the two control cell values around value 1 (Figure 9D). Interestingly, EGFR mRNA levels followed the CCHCR1 expression pattern rather than Ki67 expression pattern (Figure 9C,E). By forcing HaCaT cells to differentiate with high-calcium medium, the expression of CCHCR1 remained unaltered as measured with TaqMan PCR (data not shown), agreeing with our previous data that CCHCR1 mRNA levels were non-altered in differentiated normal KCs [1]. Involucrin was used as a differentiation marker to confirm the differentiation status of the HaCaT cells upon calcium administration [15].

## Discussion

Our results presented here show that CCHCR1, a candidate gene for psoriasis, was expressed in the majority of SCCs, BCCs and KAs studied, suggesting that CCHCR1 may have a role not only in KC proliferation but also in transformation. EGFR expression correlated with CCHCR1 expression, and cyclin-D1, a downstream target of EGFR and a positive regulator of cell cycle progression [16], was also found expressed in the same areas. In normal epidermis, both EGFR and CCHCR1 are expressed basally [4], [8] (Figure 1). Expression of EGFR, “a survival factor for tumor cells”, is even considered to be necessary to maintain KCs in a proliferative state [8]. We postulate that EGFR may regulate CCHCR1 expression as we have recently demonstrated that EGF stimulation upregulates CCHCR1 protein expression [2]. Our experiments here with transfected HaCaT cells suggested that overexpression of CCHCR1 does not regulate EGFR expression.

According to our findings, transformed KCs have a different CCHCR1 status in correlation to the hyperproliferation marker Ki67 status as compared with non-tumorigenic KCs. Distinct from the benign hyperproliferative disorder psoriasis ([1]; Figure 6E–F), expression of the hyperproliferation marker Ki67 was demonstrated here in the same areas as CCHCR1 expression at the invasive front of SCCs. We have previously shown that in breast and lung adenocarcinomas, CCHCR1 positive cells are Ki67 negative [1] but this discrepancy may be due to differences between adenocarcinoma and SCC cells or the site organ of cancer. The positive correlation with CCHCR1 and Ki67 expression in skin cancers was supported by the expression profiling of cutaneous SCC cell lines. However, there was negative correlation with CCHCR1 and EGFR expression in these same cell lines as well as in invasive FaDu tumor cells [17], suggesting that EGFR transcription was inhibited. However, this might reflect different EGFR variants studied, since the specific probe for variant 2 seemed to be more downregulated than variant 1 or probes recognizing all variants.

Despite positive CCHCR1 and Ki67 expression in vivo in skin cancer and in Affymetrix assay of SCC cell lines, we show that CCHCR1 and Ki67 mRNAs are downregulated in cultured cells with ascending transformation and also in non-tumorigenic HaCaT cells treated with compounds that promote tumors in vivo. Metastatic RT3 and invasive II4 cells expressed CCHCR1 and Ki67 less than tumorigenic A5 and immortalized HaCaT cells, suggesting that CCHCR1 and Ki67 expression increases inversely to the level of ras-transformation. Similarly, EGFR expression was downregulated in all ras-transformed cells, even in A5 cells. CCHCR1 mRNA was also diminished in the invasive FaDu cells compared to A431 correlating with up-regulation of EGFR from FaDu to A431 cells. High EGFR expression in A431 cells compared with FaDu cells agrees with previous studies [17]. Furthermore, in SCCs, the CCHCR1 immunostaining was heterogeneous in atypic cells – many atypic cells were also devoid of Ki67.

The tumor promoters OA and menadione downregulated the expression of CCHCR1, Ki67, and EGFR mRNAs in HaCaT cells. OA inhibits serine/threonine-specific protein phosphatases, including protein phosphatases 1, 2A, and PP3 [18] and activates cellular Erk1/2, JNK and p38 MAPK signaling pathways and AP-1 transcription factors [19]–[21] in addition to activating Akt-1, a pro-survival serine-threonine kinase [22]. Menadione inhibits protein tyrosine phosphatases activating ErbB2 which is overexpressed in BCC and downregulated in SCC relative to normal epidermis [23]. Interestingly, EGFR activity has been implicated in OA-induced carcinogenesis and menadione-induced ErbB2 activation [24], [25]. Furthermore, EGFR has been reported to activate Erk1/2 and Akt in SCC [10].

Ultraviolet radiation is one of the most important factors predisposing to skin cancer and is also known to activate EGFR [9]. We have not detected significant changes in CCHCR1 mRNA levels in HaCaT cells cultured for various periods after UVA/UVB radiation (Suomela, Latonen and Saarialho-Kere, unpublished data). We could not demonstrate any effect of H_2_O_2_ (producing oxidative stress) on CCHCR1 mRNA expression either, although also OA as well as menadione are known to induce the formation of reactive oxygen species and lipid peroxidation on immortalized cell lines [26], [27], suggesting that oxidative stress was not involved in the interaction of OA and CCHCR1.

Finally, we demonstrate that in HaCaT cells, CCHCR1 expression decreased with proliferation, in accordance with our earlier reports [1], [3], [4]. After provoking proliferation in HaCaT cells as described earlier [14], the more proliferative the non-tumorigenic HaCaT cells were, the less CCHCR1 was expressed. Interestingly, EGFR mRNA expression followed that of CCHCR1 mRNA expression rather than Ki67 expression, suggesting a common up-stream effector in signaling pathways. We cannot, however, in this setting exclude the effect of cell confluency alone on CCHCR1 expression as it affects also the proliferation of HaCaT cells.

Malignant transformation of psoriatic lesional KCs is very uncommon despite of uncontrolled KC proliferation and co-existence of chronic inflammation. The reason for protection from cancer development remains unclear. Reduced AP-1 levels in psoriatic KCs have been suggested as an explanation [28], [29]. AP-1 mediated pathway is also involved in EGFR activation leading to KC hyperproliferation [5]. When the CCHCR1 transgenic risk mice, with downregulated vitamin D receptor [30], were wounded and PMA-treated, KC proliferation was reduced [3], suggesting less active AP-1 or STAT3 signaling. Furthermore, vitamin D, a drug used in the treatment of psoriasis, downregulates EGFR and cyclin-D1 [31]. CCHCR1 has also been proposed to regulate migration of the RNA polymerase II subunit 3 (RPB3) [32], that activates the activating transcription factor 4 (ATF4 or CREB2) [31] associating with growth arrest [34], [35] and being able to form heterodimers with members of the AP-1 family. The present study implicates that CCHCR1, a candidate gene for psoriasis, has a function in KC biology also in malignant transformation. Further studies are needed to reveal the complex signaling system behind the herein demonstrated possible connections with CCHCR1 and EGFR network in KC proliferation and transformation.

In conclusion, CCHCR1 is upregulated in SCC in vivo and is present in EGFR-positive cells. Its expression was not induced in vitro in the most aggressive and metastatic SCC cell lines. Ascending tumorigenicity and proliferative state downregulated CCHCR1 mRNA in HaCaT cells, agreeing with our previous results on CCHCR1 as an antiproliferative agent. Furthermore, CCHCR1 and Ki67 expression correlated in SCC samples in vivo and in human SCC cell lines by Affymetrix and TaqMan assay. Previously, we could not observe correlation with CCHCR1 and Ki67 in psoriasis lesions [4] nor in PMA-induced hyperproliferation of transgenic mouse skin [30], and analogously in the current study, we found negative correlation in the proliferation assay of benign HaCaT cells. Thus, in contrast to benign KC hyperproliferation in psoriasis, Ki67 expression in vivo and in vitro associated with CCHCR1 expression in malignant transformation. We hypothesize that in psoriasis dysregulation of CCHCR1 may lead to abnormal KC hyperproliferation [3], whereas it may participate in regulating cell proliferation in the early stages of KC transformation as well, but beyond a certain point in oncogenesis CCHCR1 cannot control this phenomenon any longer.

## Materials and Methods

### Chemicals and materials

Cell culture media and fetal calf serum were from GIBCO Invitrogen Life Technologies (Paisley, Scotland). Cytokines and chemicals were from Calbiochem (La Jolla, CA), Invitrogen Life Technologies, Merck (Darmstadt, Germany), R&D Systems (Abingdon, UK), Roche Molecular Biochemicals (Indianapolis, IN), and Sigma (St. Louis, MO). Reagents for real-time quantitative PCR were from Applied Biosystems (Warrington, UK).

### Cell cultures

The immortalized human KC cell line HaCaT and its subclones with increasing malignancy A5, II4, and RT3 as well as A431 and FaDu SCC cell lines were cultured as described earlier [31], [36], [37]. To study the regulation of CCHCR1 expression, HaCaT cells were plated on tissue culture plates and allowed to reach 70–80% confluence. Then the cells were depleted of serum overnight prior to stimulation with anisomycin (25 ng/ml; 6, 24, and 48 h), menadione (10 µM; 6 h), OA (10 nM and 30 nM; 24 h), PMA (10 ng/ml, 25 ng/ml and 50 ng/ml; 24 h), staurosporine (25 nM, 24 h), IL-6 (1 ng/ml, 20 ng/ml; 24 and 48 h), staphylococcal endotoxin B (0.1 ng/ml, 1 ng/ml, 10 ng/ml, 100 ng/ml; 24 and 48 h), and tamoxifen (50 nM, 100 nM, 1000 nM; 24 and 48 h) (all from Sigma). Activin A (18 ng/ml; 6, 24, and 48 h) was from R&D Systems (Minneapolis, MN) and leptin (10 ng/ml, 100 ng/ml, 1000 ng/ml; 30 min, 60 min and 24 h) from Calbiochem. Cells were also stimulated by H_2_O_2_ (0.05 mM, 0.1 mM, 0.5 mM, 1.0 mM; 2 and 4 h). The cells were lyzed and total RNA was extracted using RNeasy Mini-kit (Qiagen, Chatsworth, CA) as instructed by the manufacturer. Cells given fresh serum-free medium were used as controls. All treatments were carried out in triplicate, and the results were confirmed in at least two independent experiments.

Human cutaneous SCC cell lines (n = 8) were established at the time of operation from five primary and three metastatic SCCs [38]. Cells were cultured in DMEM supplemented with 6 nmol/l glutamine, nonessential amino acids, and 10% fetal calf serum (FCS). Normal human epidermal KC cell lines (n = 5) were established from skin samples, and one NHEK cell lines was purchased from PromoCell (Heidelberg, Germany). The cell lines were cultured in Keratinocyte Basal Medium® 2, supplemented with SingleQuots® (Cambrex Bioscience; Walkersville, MD), as previously described [39]. The biopsies for cell lines were approved by the Joint Ethical Committee of the University of Turku and Turku University Central Hospital. Participants gave their informed consent, and the study was conducted according to Declaration of Helsinki.

### Quantitative Real-Time PCR (TaqMan RT-PCR)

cDNA synthesis and TaqMan RT-PCR for CCHCR1, EGFR, Ki67, and GAPDH were performed as described earlier [1], [40]. Reactions were performed with the Applied Biosystems 7500 Fast Real-Time PCR System with standard protocol. Quantitative PCR reactions to confirm microarray results were performed using the ABI 7900HT real-time PCR machine (Applied Biosystems), with each reaction containing 4 ng of reverse transcribed RNA in a 20 µl reaction. EGFR and Ki67 primers and probes were purchased from Applied Biosystems (Hs01076092_ml and Hs01032443_ml, respectively). The relative RNA levels in each sample were determined by performing standard curves for all target genes covering 0.1–10 ng of RNA. Each sample was run in duplicate and β-actin was used as a control to normalize for differences in the amount of total RNA in each sample.

### Proliferation assays

Proliferation of HaCaT cells was assayed according to Pivarcsi et al. [14]. Shortly, HaCaT cells were synchronized by culturing in DMEM (Gibco Invitrogen Life Technologies) containing 5% fetal bovine serum for five days after confluence was reached. The cells were washed and medium replaced by serum-free DMEM. After culturing for one week, cells were trypsinized, counted and their viability determined by trypan blue staining. They were seeded into 25-cm^2^ culture flasks at a density of 5×10^3^ cells/cm^2^ in 5% FBS-DMEM to induce the proliferation. Samples for TaqMan RT-PCR analysis were taken at different time points and the experiment was repeated three times.

### Differentiation assay

HaCaT keratinocytes were cultured in defined K-SFM (GIBCO Invitrogen Life Technologies) medium of low calcium concentration (<1×10^−4^ M). After five days, cells were trypsinized and seeded onto 25-cm^2^ culture flasks at a density of 1×10^4^ cells/cm^2^ using the same medium as above except high calcium concentration (1.8×10^−3^ M) to promote cell differentiation [41]. Samples for TaqMan RT-PCR analysis were taken at different time points.

### Tissue samples

Paraffin-embedded, formalin-fixed samples of human skin were obtained from the Department of Dermatopathology, Skin and Allergy Hospital, University of Helsinki, Finland and they were cut at the thickness of 4 µm. To study CCHCR1 expression in relation to tumorigenesis the following specimens were selected: KA (n = 18), AK (n = 11), Bowen's disease (n = 21), SCCs (n = 22, of which grade I n = 9, grade II n = 6, grade III n = 7), and BCCs (n = 15, of which morpheaform n = 3, superficial n = 3, nodular n = 9). The study was conducted in accordance to the Declaration of Helsinki principles and use of anonymous archival samples without written consent was approved by the corresponding Ethical Review Board of Helsinki University Central Hospital, Helsinki, Finland.

### Immunohistochemistry

Immunostainings of tissue sections were performed using the avidin-biotin-peroxidase complex method (Vectastain ABC Kit, Vector Laboratories, Burlingame, CA, for CCHCR1 and cyclin-D1; or StreptABComplex/HRP Duet (Mouse/Rabbit), DAKO, A/S Glostrup, Denmark, no. K0492 for EGFR and Ki67). Samples were immunostained using antibodies for Ki67 (DakoCytomation, A/S Glostrup, Denmark), EGFR (Zymed Laboratories, San Francisco, CA), cyclin-D1 (Thermo Fisher Scientific, Fremont, CA), and CCHCR1 [1], [4]. Sections were pretreated with trypsin (10 mg/ml; CCHCR1, EGFR) or by microwaving in citrate buffer (Ki67, cyclin-D1). Aminoethylcarbazole or diaminobenzidine were used as chromogenic substrates and hematoxylin as counterstain. Controls with preimmune sera or normal rabbit immunoglobulin were used as negative controls.

### Microarray analyses

Total RNA was extracted from cultured cells using TRIzol reagent (Invitrogen; Carlsbad, CA) or using the Qiagen RNeasy kit (Qiagen). Total RNA was reverse-transcribed and used as a template for biotin-labeled cRNA. Samples were hybridized to Affymetrix's Human Genome U133 Plus 2.0 array, and the data analyses were performed at the Microarray Centre of Turku Centre for Biotechnology according to the manufacturer's protocol. Sequence specificity of Affymetrix probes was verified by BLAST search. EGFR 211607_x_at and EGFR 210984_x_at recognize all variants, EGFR 211551_at aberrant EGFR, EGFR 211550_at variant 4, EGFR 201984_s_at and EGFR 201983_s_at variant 1, EGFR 1565484_x_at and EGFR 1565483_at variant 2. CCHCR probes recognize all variants and MKi67 probes recognize the same transcript.

### Immunofluoresence staining of transfected cells

HaCaT cells were transiently transfected with the pCMV5-CCHCR1 construct [4], [42], [30] using the FuGENE HD reagent (Roche Diagnostics) according to the manufacturers' instructions. Cells were fixed with 4% paraformaldehyde-PBS solution 24 hours after transfection, washed with PBS and permeabilized with 0.5% Triton-X100. Cells were immunostained with the rabbit polyclonal antibody for CCHCR1 [4] and mouse monoclonal antibody against the EGFR (Zymed laboratories, Invitrogen immunodetection). Alexa Fluor 555-anti mouse and Alexa Fluor 488-anti rabbit IgGs (Invitrogen) were used as secondary antibodies. Cell nuclei were visualized with 4′-6-Diamidino-2-phenylindole staining.

### Statistical analysis

Statistical significance of the differences in mRNA expression levels in TaqMan analyses was calculated using the unequal, two-tailed, odd t-test or using the non-parametric Mann-Whitney U test. Correlation was calculated with Pearson's correlation coefficient.

## Supporting Information

Figure S1 Expression of CCHCR1 in grade III SCC. CCHCR1 protein is expressed in proliferative cancer cells at the invasive front of grade III SCC. Scale bar: 125 µm
(4.00 MB TIF)
