# Supplementary figures and images for: CCHCR1 Is Up-Regulated in Skin Cancer and Associated with EGFR Expression
Source: PLoS One. 2009 Jun 24;4(6):e6030. doi: 10.1371/journal.pone.0006030 (PMC2696036; doi:10.1371/journal.pone.0006030)

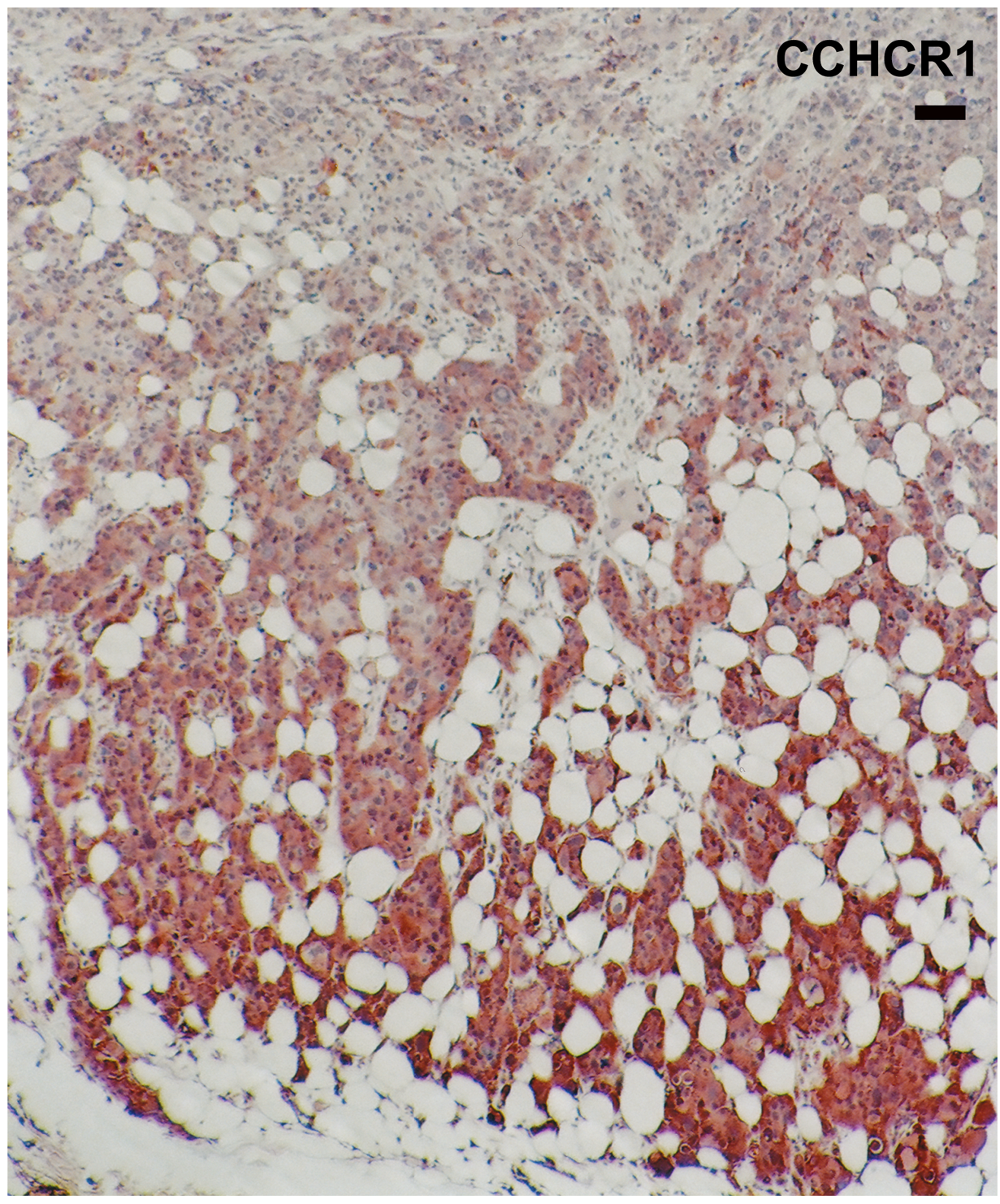

Supplement: Figure S1 — Expression of CCHCR1 in grade III SCC. CCHCR1 protein is expressed in proliferative cancer cells at the invasive front of grade III SCC. Scale bar: 125 µm (4.00 MB TIF) [file pone.0006030.s001.tif]
